# Association Between Eating Speed and Metabolic Syndrome in a Three-Year Population-Based Cohort Study

**DOI:** 10.2188/jea.JE20140131

**Published:** 2015-04-05

**Authors:** Bing Zhu, Yasuo Haruyama, Takashi Muto, Takako Yamazaki

**Affiliations:** 1 Dept. of Health Education, Anhui Provincial Center for Disease Control and Prevention, Hefei, China; 1 中国安微省疾病控制中心; 2 Dept. of Public Health, Dokkyo Medical University School of Medicine, Mibu-machi, Shimotsuga-gun, Tochigi, Japan; 2 獨協医科大学医学部公衆衛生学講座; 3 Division of Health Promotion, Health Centre of Soka City, Soka, Saitama, Japan; 3 埼玉県草加市保健センター

**Keywords:** eating speed, metabolic syndrome, cohort study, risk factor, epidemiology

## Abstract

**Background:**

Metabolic syndrome has received increased global attention over the past few years. Eating behaviors, particularly eating speed, have long been of interest as factors that contribute to the development of obesity and diabetes. The aim of this study was to assess the relationship between eating speed and incidence of metabolic syndrome among middle-aged and elderly Japanese people.

**Methods:**

A total of 8941 community residents from Soka City in Saitama Prefecture, aged from 40 to 75 years and without a diagnosis of metabolic syndrome, participated in the baseline survey in 2008 and were followed until 2011. Anthropometric measurements and lifestyle factors were measured at baseline and follow-up. The association between eating speed and incidence of metabolic syndrome was evaluated using Cox proportional hazards models adjusted for potential confounding variables.

**Results:**

During the 3-year follow-up, 647 people were diagnosed with metabolic syndrome (25.0 cases/1000 person-years). The incidence rates of metabolic syndrome among non-fast-eating and fast-eating participants were 2.3% and 3.1%, respectively. The multivariate-adjusted hazard ratio for incidence of metabolic syndrome in the fast-eating group compared to the not-fast-eating group was 1.30 (95% confidence interval [CI], 1.05–1.60) after adjustment for the potential confounding factors. Eating speed was significantly correlated with waist circumference and high-density lipoprotein cholesterol (HDL-C) components of metabolic risk factors. Hazard ratios in the fast-eating group compared with the reference group were 1.35 (95% CI, 1.10–1.66) for waist circumference and 1.37 (95% CI, 1.12–1.67) for HDL-C.

**Conclusions:**

Eating speed was associated with the incidence of metabolic syndrome. Eating slowly is therefore suggested to be an important lifestyle factor for preventing metabolic syndrome among the Japanese.

## Abstract (ja)

**背景：:**

近年、メタボリックシンドローム（MetS）が世界的に注目されている。食習慣、特に食べる速さは肥満と糖尿病の発生につながる因子の一つとして多くの関心を集めている。本研究は、日本の中高年齢者における食べる速さとMetS罹患率の関連を明らかにすることを目的とした。

**方法：:**

2008年にベースライン調査を受けた埼玉県草加市の住民の中、MetSを除外した40歳から75歳までの8,941人を2011年まで追跡した。検査項目及びライフスタイルはベースラインとフォローアップ時に測定した。分析は潜在的な交絡因子を調整したコックス比例ハザードモデルを用いて、食べる速さとMetS罹患率との関連を検討した。

**結果：:**

追跡した３年の間では647名がMetS（25.0/1000人年）と診断された。食べるが速くない群と速い群のMetS罹患率はそれぞれ2.3%と3.1%で、潜在的な交絡因子を調整したハザード比（95%CI）は1.00（参照）と1.30（1.05-1.60）であった。また、食べる速さと腹囲やHDLコレステロールとの関連が認められ、参照群に比べて食べる速さが速い群のハザート比（95％CI）はそれぞれ1.35（1.10-1.66）と1.37（1.12-1.67）で有意に髙かった。

**結論：:**

食べる速さはMetS罹患率との関連が認められた。ゆっくり食べることは日本人のMetSを防止するための重要なライフスタイルであることが示唆された。

## INTRODUCTION

Metabolic syndrome has received increased global attention over the past few years.^1^^–^^5^ Metabolic syndrome is a constellation of interrelated risk factors of metabolic origin (metabolic risk factors) that appear to directly promote the development of atherosclerotic cardiovascular disease (ASCVD).^6^ Research has indicated that metabolic syndrome is a major determinant of ischemic heart disease and stroke among middle-aged Japanese men and women.^7^ Patients with metabolic syndrome also have an increased risk of developing type 2 diabetes.^8^

Eating behaviors, particularly eating speed, have long been of interest as factors that contribute to the development of obesity and diabetes. Recent epidemiological cross-sectional and longitudinal studies have shown that eating speed is associated with obesity and diabetes.^9^^–^^22^ However, to our knowledge, only one study has reported that the eating rate in severely obese women and men may be a determinant of metabolic syndrome.^23^ No cohort studies have focused on the relationship between eating speed and incidence of metabolic syndrome.

In this article, we assessed the relationship between eating speed and incidence of metabolic syndrome among middle-aged and elderly Japanese people in a three-year cohort study. The hypothesis was that eating speed is a risk factor in predicting metabolic syndrome.

## METHODS

### Participants

The study participants were community residents of Soka City in Saitama Prefecture, Japan, which is a city with a population of about 233 000. The study design has been previously reported.^24^^,^^25^ In 2008, a total of 8958 community residents aged from 40 to 75 years old without a diagnosis of metabolic syndrome participated in the baseline survey. We excluded 17 residents because they were missing data on eating speed. Therefore, 8941 participants were enrolled in the present study and followed until 2011.

### Measurements

The baseline and follow-up survey included a medical history, physical examination, anthropometric measurements, and a questionnaire regarding lifestyle behaviors.

All of the measurements were provided by medical institutions. Body weight and height were measured with no shoes and excess clothing removed on the same calibrated scale at the baseline and follow-up. BMI was calculated as the body weight (kg) divided by the square of the height (m^2^). Waist circumference was measured by nurses. Systolic blood pressure (SBP) and diastolic blood pressure (DBP) were measured using auto-manometers (Omron Co., Tokyo, Japan). Fasting blood samples were obtained from all subjects, and triglycerides (TG), low-density lipoprotein cholesterol (LDL-C), high-density lipoprotein cholesterol (HDL-C), and glycated hemoglobin (HbA1c) were measured at a laboratory (Saitama, Japan).

Information on lifestyle factors, such as smoking, drinking alcohol, dietary behaviors, physical activity, and sleeping condition, as well as medical history, were obtained by a self-administered questionnaire at the baseline and follow-up. Current smokers were defined as those who had been smoking for at least 6 months or had smoked over 100 cigarettes and were still smoking in the previous month. Drinking alcohol was indicated by the frequency of drinking and the amount of alcohol consumed per day.^25^ Dietary behaviors included eating speed and frequency of eating dinner late, eating snacks, and skipping breakfast. Physical activity included regular exercise and daily physical activity. The medical history included questions regarding history of hypertension or taking hypertension medication, history of dyslipidemia or taking lipid-lowering medication, and history of diabetes or taking diabetes medication.

In the questionnaire, the speed of eating was self-reported by the answer to the question: “How fast is your rate of eating (speed of eating)”, chosen from three semi-quantitative categories: “slow”, “medium”, and “fast”.

### Diagnosis of metabolic syndrome

Metabolic syndrome was diagnosed according to the National Cholesterol Education Program Adult Treatment Panel III (NCEP-ATP III).^6^ A participant was deemed to have metabolic syndrome when three or more of the following criteria were satisfied: (1) waist circumference >102 cm in men or >88 cm in women; (2) HDL cholesterol <40 mg/dL in men or <50 mg/dL in women; (3) triglyceride level ≥150 mg/dL; (4) systolic blood pressure >130 mm Hg or diastolic blood pressure >85 mm Hg; (5) blood glucose level ≥110 mg/dL or HbA1c ≥5.6% according to the Japanese Diabetes Society. In the present study, time diagnosed with metabolic syndrome was counted from the year at which the health check-up was undergone. When the health check-up was not undergone for the first year after baseline, it was considered a censored case.

### Statistical analysis

Since the number of participants who reported their speed of eating as “slow” was low (*n* = 687, accounting for 7.7%), we divided the participants into two eating-speed categories as follows: not fast (slow and medium) and fast. Baseline characteristics between the two eating-speed categories were analyzed using Student’s *t*-test for continuous variables, the Mann-Whitney test for nonparametric variables, and the Chi-squared test for categorical variables. Person-years were calculated as the sum of individual follow-up times until the occurrence of metabolic syndrome, censoring, or the end of 2011. The associations between eating speed and incidence of metabolic syndrome and each component of metabolic syndrome were evaluated using Cox proportional hazards models adjusted for multiple variables (age, sex, smoking, drinking alcohol, dietary behavior, physical activity, sleeping, and medication history).

### Ethical consideration

Data were received from the Health Centre of Soka City with complete anonymity. Thus, informed consent was not obtained from participants during data collection. This cohort study was in compliance with the ethical guidelines for epidemiological research of the Ministry of Education, Culture, Sports, Science and Technology and the Ministry of Health, Labour and Welfare of Japan,^26^ and ethical approval was given by the ethics committee of Dokkyo Medical University (No. 2057).

## RESULTS

### Baseline examination

Baseline characteristics of study participants in the eating-speed categories are shown in Table 1. The mean age of participants was 63.7 years, and 61.7% were female. The participants who ate faster were younger and more likely to be male, with a higher BMI, larger waist circumference, higher TG and HbA1c, and lower HDL-C. Participants who were inclined to eat faster were also more likely to be current smokers, to often eat dinner later (over 3 times per week), to often eat snacks (over 3 times per week), to often skip breakfast (over 3 times per week), to drink less than 22 g of alcohol per day, and to have a history of taking medication for hypertension, dyslipidemia, and diabetes.

**Table 1.** Baseline characteristics of study participants by eating speed

Baseline	All (*n* = 8941)	Eating speed		*P*-value^a^

		Not fast (*n* = 7040)	Fast (*n* = 1901)	
Age, years	63.7 (7.9)	64.1 (7.7)	62.5 (8.4)	<0.001
Height, cm	157 (8.5)	156.6 (8.3)	158.8 (8.8)	<0.001
Weight, kg	56.4 (10.1)	55.5 (9.6)	60.1 (11.1)	<0.001
BMI, kg/m^2^	22.8 (3.1)	22.6 (2.97)	23.7 (3.3)	<0.001
WC, cm	82 (8.8)	81.4 (8.6)	84.2 (9.3)	<0.001
SBP, mm Hg	130.9 (15.7)	131.0 (15.8)	130.5 (15.7)	0.214
DBP, mm Hg	76.8 (9.6)	76.8 (9.6)	77.0 (9.6)	0.453
TG, mg/dL^b^	116.1 (77.6)	114.0 (74.8)	123.6 (86.8)	<0.001
HDL-C, mg/dL	65.4 (16.7)	65.8 (16.7)	63.7 (16.6)	<0.001
LDL-C, mg/dL	126.9 (31.5)	126.9 (31.8)	126.9 (30.3)	0.985
HbA1c, %	5.3 (0.6)	5.2 (0.6)	5.3 (0.6)	0.019
Female sex, %	61.7	63.4	55.7	<0.001
Current smoker, %	17.8	16.9	21.1	<0.001
Dietary behaviors				
Eating dinner late, %	20.0	18.8	24.4	<0.001
Eating snacks often, %	10.4	9.4	14.1	<0.001
Skipping breakfast often, %	9.7	9.1	11.8	<0.001
Physical activity				
Regular exercise, %	45.0	44.7	45.9	0.333
Daily physical activity, %	52.2	52.6	50.6	0.106
Drinking alcohol				
Drinking every day, %	51.4	51.9	49.7	0.096
Drinking <22 g each time, %	63.5	64.7	59.1	<0.001
Sleeping well, %	76.4	77.3	73.1	<0.001
Medication history				
Medication history of BP, %	29.4	28.5	32.7	<0.001
Medication history for glucose, %	4.3	3.9	5.4	0.007
Medication history for lipids, %	15.7	15.2	17.6	0.009

BMI, body mass index; BP, blood pressure; DBP, diastolic blood pressure; HbA1c, glycated hemoglobin; HDL-C, high-density lipoprotein cholesterol; LDL-C, low-density lipoprotein cholesterol; SBP, systolic blood pressure; TG, triglycerides; WC, waist circumference.

Data are reported as mean (standard deviation) or %.

^a^By chi-squared or Student’s *t*-test.

^b^By Mann-Whitney test.

### Follow-up study

#### Association between eating speed and metabolic syndrome

The incidence rate of metabolic syndrome after three years of follow-up is shown in Table 2. During the 3-year follow-up, 647 people were diagnosed with metabolic syndrome (25 cases/1000 person-years). The crude incidence rates of metabolic syndrome in the eating-speed categories (not fast and fast) were 2.3% and 3.1%, respectively (Table 3). In age- and sex-adjusted analysis, eating speed was significantly correlated with incidence of metabolic syndrome (hazard ratio 1.40; 95% confidence interval [CI], 1.18–1.67). The fully multivariate-adjusted hazard ratio, adjusted for age, sex, smoking, drinking alcohol, dietary behavior, physical activity, sleeping, and medication history, for incidence of metabolic syndrome in the fast-eating group compared to the not-fast-eating group was 1.30 (95% CI, 1.05–1.60).

**Table 2.** Incidence rate of metabolic syndrome after follow-up

	MetS	Person-years	Incidence rate (%)
Total	647	25 817.5	2.5
Males	215	9926.5	2.2
Females	432	15 891.0	2.7

MetS, metabolic syndrome.

**Table 3.** Adjusted hazard ratio of incidence of metabolic syndrome by eating speed

	Eating speed	

	Not fast	Fast
*n*	7040	1901
Number of incident cases	477	170
Person-years of follow-up	20 383.5	5434
Incidence rate	2.3%	3.1%
Hazard ratio (95% CI)		
Model 1^a^	1.00 (reference)	1.40 (1.18–1.67)
Model 2^b^	1.00 (reference)	1.30 (1.05–1.60)

^a^By the Cox proportional hazard model, adjusted for age and sex.

^b^By the Cox proportional hazard model, adjusted for age, sex, smoking, drinking alcohol, dietary behavior, physical activity, sleeping, and medication history.

#### Association between eating speed and each component of metabolic syndrome

Table 4 shows the adjusted hazard ratio of each item of metabolic syndrome according to categories of eating speed. Eating speed was significantly correlated with waist circumference and HDL-C. Hazard ratios for the fast-eating group, adjusted for age, sex, smoking, drinking alcohol, dietary behavior, physical activity, sleeping, and medication history, were 1.35 (95% CI, 1.10–1.66) for waist circumference and 1.37 (95% CI, 1.12–1.67) for HDL-C. No significant correlations were found between the three other components of metabolic syndrome (TG, blood pressure, and blood glucose) and eating speed.

**Table 4.** Adjusted hazard ratio of each item of metabolic syndrome by eating speed

	WC		HDL-C		TG		BP		BG	
				
	Eating speed		Eating speed		Eating speed		Eating speed		Eating speed	
				
	Not fast	Fast	Not fast	Fast	Not fast	Fast	Not fast	Fast	Not fast	Fast
*n*	6545	1718	6775	1863	5803	1526	6359	1789	5381	1400
Number of incident cases	604	176	540	189	1205	373	1013	305	554	144
Person-years of follow-up	18 574	4837	19 384	5258.5	15 397.5	3952.5	17 372.5	4873.5	15 531	4051
Incidence rate	3.3%	3.6%	2.8%	3.6%	7.8%	9.4%	5.8%	6.3%	3.6%	3.6%
Hazard ratio (95% CI)										
Model 1^a^	1.00	1.42 (1.20–1.68)	1.00	1.36 (1.15–1.60)	1.00	1.20 (1.07–1.35)	1.00	1.08 (0.95–1.23)	1.00	1.02 (0.85–1.23)
Model 2^b^	1.00	1.35 (1.10–1.66)	1.00	1.37 (1.12–1.67)	1.00	1.15 (0.99–1.31)	1.00	1.10 (0.94–1.27)	1.00	0.93 (0.75–1.16)

BG, blood glucose; BP, blood pressure; HDL-C, high-density lipoprotein cholesterol; TG, triglycerides; WC, waist circumference.

^a^By the Cox proportional hazard model, adjusted for age and sex.

^b^By the Cox proportional hazard model, adjusted for age, sex, smoking, drinking alcohol, dietary behavior, physical activity, sleeping, and medication history.

## DISCUSSION

To our knowledge, this is the first cohort study to examine the association between metabolic syndrome and the eating speed. Furthermore, we explored the relationships between each component of metabolic syndrome and eating speed in this study. Our results suggest that a faster eating speed may increase the incidence of metabolic syndrome, especially in association with a larger waist circumference and higher HDL-C levels.

The results were consistent with previous cross-sectional and cohort studies. Sakurai et al found that eating speed was associated with the incidence of type 2 diabetes mellitus in middle-aged Japanese men.^20^ Multivariate-adjusted hazard ratios across the categories of eating speed (slow, medium, and fast) were 1.00 (reference), 1.68 (95% CI, 0.93–3.02), and 1.97 (95% CI, 1.10–3.55), respectively. Hsieh et al indicated that eating fast increases metabolic risk factors in Japanese men and women among residents who underwent health examinations.^21^ Maruyama et al also reported that eating speed was associated with being overweight in Japanese men and women.^22^ The multivariable adjusted odds ratio of being overweight for eating quickly was 1.84 (95% CI, 1.42–2.38) for men and 2.09 (95% CI, 1.69–2.59) for women.

There are several hypotheses to explain why eating speed can have an impact on the incidence of metabolic syndrome. First, due to a lack of satiety, rapid ingestion may cause overeating before the stomach senses fullness.^9^ Second, eating fast may lead to insulin resistance,^19^ resulting in metabolic syndrome.

The strength of the present investigation is that it involved a three-year cohort study, which can support a causative role of eating speed in the incidence of metabolic syndrome to a certain extent, unlike previous cross-sectional studies. Also, the study used a large sample recruited from community residents, unlike the samples of adolescents and occupational workers in previous studies. This improves the generalizability of the findings. Finally, our study analyzed the association between each component of metabolic syndrome and eating speed, which can generate more specific information for future intervention studies.

The limitations of our study should also be noted. First, eating speed was subjectively self-reported by study subjects, so we cannot exclude reporting bias. We used only 2 categories of eating speed (‘slow and medium’ and ‘fast’) due to the small sample size of one category. Validation studies of the self-reported speed of eating in comparison with careful quantified measurements of eating rate would be useful. Another limitation is that we did not control for energy intake. Since the relationship between eating behavior and metabolic syndrome concerns the total energy intake, future studies should focus on how eating speed impacts the incidence of metabolic syndrome after controlling for the total energy intake.

In conclusion, eating speed was associated with the incidence of metabolic syndrome, since the associations were still significant after adjusting for other lifestyle factors. Eating slowly may be an important lifestyle factor for the prevention of metabolic syndrome among Japanese.

## ONLINE ONLY MATERIAL

Abstract in Japanese.
