# Supplementary material for: Association Between Eating Speed and Metabolic Syndrome in a Three-Year Population-Based Cohort Study
Source: J Epidemiol. 2015 Apr 5;25(4):332–6. doi: 10.2188/jea.JE20140131 (PMC4375288; doi:10.2188/jea.JE20140131)
Supplement: Abstract in Japanese. [file je-25-332-s001.pdf]

地域住民対象の3年コホート研究における食べる速さとメタボリックシンドロームとの関連

朱氷[1 2]、春山康夫[2*]、武藤孝司[2]、山崎章子[3]
[1]中国安徽省疾病控制中心、[2]獨協医科大学医学部公衆衛生学講座、[3]埼玉県草加市保健センター

背景：近年、メタボリックシンドローム（MetS）が世界的に注目されている。食習慣、特に食べる速さは肥満と糖尿病の発生につながる因子の一つとして多くの関心を集めている。本研究は、日本の中高年齢者における食べる速さと MetS 罹患率の関連を明らかにすることを目的とした。
方法：2008 年にベースライン調査を受けた埼玉県草加市の住民の中、MetS を除外した 40 歳から 75 歳までの 8,941 人を 2011 年まで追跡した。検査項目及びライフスタイルはベースラインとフォローアップ時に測定した。分析は潜在的な交絡因子を調整したコックス比例ハザードモデルを用いて、食べる速さと MetS 罹患率との関連を検討した。
結果：追跡した 3 年の間では 647 名が MetS（25.0/1000 人年）と診断された。食べるが速くない群と速い群の MetS 罹患率はそれぞれ 2.3%と 3.1%で、潜在的な交絡因子を調整したハザード比（95%CI）は 1.00（参照）と 1.30（1.05-1.60）であった。また、食べる速さと腹囲や HDL コレステロールとの関連が認められ、参照群に比べて食べる速さが速い群のハザート比（95%CI）はそれぞれ 1.35（1.10-1.66）と 1.37（1.12-1.67）で有意に高かった。
結論：食べる速さは MetS 罹患率との関連が認められた。ゆっくり食べることは日本人の MetS を防止するための重要なライフスタイルであることが示唆された。

キーワード：食べる速さ、メタボリックシンドローム、コホート研究、危険因子、疫学
